# Process-Based Crop Modeling for High Applicability with Attention Mechanism and Multitask Decoders

**DOI:** 10.34133/plantphenomics.0035

**Published:** 2023-04-12

**Authors:** Taewon Moon, Dongpil Kim, Sungmin Kwon, Jung Eek Son

**Affiliations:** ^1^ Department of Agriculture, Forestry and Bioresources, Seoul National University, Seoul 08826, Republic of Korea.; ^2^ Research Institute of Agriculture and Life Sciences, Seoul National University, Seoul 08826, Republic of Korea.; ^3^ Protected Horticulture Research Institute, National Institute of Horticultural & Herbal Science, Rural Development Administration, Haman 52054, Republic of Korea.

## Abstract

Crop models have been developed for wide research purposes and scales, but they have low compatibility due to the diversity of current modeling studies. Improving model adaptability can lead to model integration. Since deep neural networks have no conventional modeling parameters, diverse input and output combinations are possible depending on model training. Despite these advantages, no process-based crop model has been tested in full deep neural network complexes. The objective of this study was to develop a process-based deep learning model for hydroponic sweet peppers. Attention mechanism and multitask learning were selected to process distinct growth factors from the environment sequence. The algorithms were modified to be suitable for the regression task of growth simulation. Cultivations were conducted twice a year for 2 years in greenhouses. The developed crop model, DeepCrop, recorded the highest modeling efficiency (= 0.76) and the lowest normalized mean squared error (= 0.18) compared to accessible crop models in the evaluation with unseen data. The t-distributed stochastic neighbor embedding distribution and the attention weights supported that DeepCrop could be analyzed in terms of cognitive ability. With the high adaptability of DeepCrop, the developed model can replace the existing crop models as a versatile tool that would reveal entangled agricultural systems with analysis of complicated information.

## Introduction

Process-based crop models have been developed and improved to support agricultural decisions on many scales and purposes [1–4]; with the process-based approach, the genotypic, environmental, and management influences on crops can be quantified. Food and feed crop models in open fields are representative process-based crop models [2], and horticultural crop models in greenhouses are also frequently reported [1,4]. These crop models have been modified and improved for decades by various research groups in various regions for diverse purposes [5–9].

Because of the variation in the crop models, they have become uncoordinated: a modification or an improvement in a crop model is not ensured for the applicability to another model. Regardless of the target crops and scales, studies on the models have redundancy problems in common [3,4,7,10]. In the decades-long course of the crop modeling progression, the methodology has been torn into pieces due to the differences in objectives and research scales, and the disjunction has resulted in more fragments and redundancy. In addition, some advanced models have been exclusively developed, so some improvements have low accessibility [4,11,12].

The fragmentation and redundancy problems are prevalent in general crop modeling studies [2,4,11]. Since the independent models have been studied in various growths, managements, and local conditions, the cause of uncertainty is rarely distinguishable [7,13]. These problems can be a cause of tardy updates in crop models. Crop models should be evaluated with multiscale conditions rather than a single season and crop for a better understanding of uncertainty [14]. Cooperation through diverse scales and fields is necessary to solve the common challenges of crop models [3,15]. Numerous improvements for food and feed crop models have been suggested based on problem recognition; however, fragmentation has not yet been solved [7,14,16–18]. Therefore, the crop model studies should overcome the fragmentation and redundancy to improve the research environment that relies on decades-old models with little change.

We selected deep learning algorithms as a potential solution to mitigate fragmentation and redundancy. Deep learning has high applicability to broad target tasks as well as remarkable abstraction ability for enormous sets of data [19–21]. With its applicability, a complicated task conducted at the enterprise became accessible with a personal computer [22]. A developed model can be adopted for heterogeneous tasks with entirely different inputs and outputs [23–25], and a core algorithm in a model can be shared regardless of the research fields [26,27]. Therefore, we expected the crop model based on deep learning to be versatile and prevalent.

The objective of this study was to develop a deep-learning-based crop model with a full deep neural network structure, DeepCrop. DeepCrop could be applied for various purposes and scales based on the applicability of deep neural networks. The development protocol included model development and evaluation processes; hence, a similar methodology can be conveniently developed, resulting in higher accessibility. From the perspective of relating the natural environment and crop growth, deep learning can be considered and used in many directions because of its high applicability [28–30].

We believe that the high applicability of DeepCrop necessitates some requirements in practice. (a) The formula and crop parameters used for the crop models contributed to the fragmentation of the model studies; therefore, the model should be constructed only with neural networks. (b) DeepCrop should be a substitute for the existing crop models, which have a purpose of the crop simulation; therefore, DeepCrop has to calculate growth changes internally, but the input for crop simulation after the model training should only be the crop environment. (c) DeepCrop should operate similar to the existing crop models to be prevalent in various ranges of the agricultural system; the developed DeepCrop can interpret sequence data with process-based calculations. (d) Since DeepCrop should show an adequate performance, DeepCrop should be competitive compared to the existing crop models. DeepCrop can be widely applied regardless of the research scales and objectives if the model achieves these requirements.

## Materials and Methods

### Cultivation and crop management

The target crop of this study was sweet peppers (*Capsicum annuum* var. *annuum*). The crops were cultivated in a Venlo-type greenhouse at the experimental farm of Seoul National University, Suwon, Korea (37.3 °N, 127.0 °E). The cultivations were conducted twice a year, and the total number was 4, under various conditions (Table 1).

**Table 1.** Differed cultivation and management conditions.

Condition	2020-1	2020-2	2021-1	2021-2
Cultivation periods	Feb 26 to Jul 7	Oct 26 to Jan 25	Mar 8 to Jul 5	Oct 23 to Jan 19
Planting density	4.08 plants/m^2^	3.06 plants/m^2^	5.95 plants/m^2^	3.06 plants/m^2^
Number of plants	96	84	65	36
Cultivar	Scirocco	Mavera & Florate	Mavera & Florate	Mavera
Topping date	Jun 15	Dec 5	-	-

Open-loop hydroponic systems with 8 cultivation gutter lines were used. Two or four cultivation lines from the total 8 lines in the system were used for the experiment. A stone wool slab and cubes (Grodan GT Master, Grodan, Roermond, the Netherlands) were used as substrates for all cultivations. Two main stems of the crops were maintained with trellis strings. Proefstation voor Bloemisterij en Glasgroente nutrient solution from the Netherlands was used for irrigation. Electrical conductivity (EC) of the nutrient solutions was maintained between 2.6 and 3.1 dS m^−1^. The fruits were harvested 2 to 3 times a week when the surfaces of the fruits were mostly colored. Four cultivation periods in 2020 to 2021 were used for the model training, validation, and test. DeepCrop was trained and validated using the 2020 data, and the sufficiently trained model was tested with the 2021 data. The 2020 data were randomly divided into the training and validation set.

### Data collection

In this study, aerial environment data were used for the crop simulations. Temperature and relative humidity were measured using a complex sensor (AQ3020, Aosong Electronics, Guangzhou, China); radiation was measured using a pyranometer (SQ-110, Apogee Instrument Inc., Logan, UT, USA). The collected data were saved on a cloud platform (ioFarm, ioCrops Inc., Seoul, Korea). The missing environmental data were interpolated using one-dimensional linear interpolation. The loss percentage of the environmental data was approximately 7%.

The growth data such as fresh weight (FW), dry weight (DW), and leaf area were collected with the destructive investigation. The investigation was conducted 5 times for each cultivation except the cultivation in the latter half of 2021. At that time, the crops were investigated only at the end of the cultivation to exclude biases from crop population decrease. For organ DW, destructive organs were dried for 72 h at 80 °C in a forced-air drying oven (HB-503LF, Hanbaek Co. Ltd., Bucheon-si, Gyeonggi-do, Korea). Since the destructive investigation varied the number of plants, the harvest data were normalized to fruits per plant using the total number of crops.

Leaf area was measured from the image using Easy Leaf Area [31]. Plant height was calculated from the higher value between summations of the node lengths of each stem. The other length factors were measured using a digital vernier caliper.

For the processed input factors, growing degree days (GDD) were calculated by daily average temperature and the base temperature (*T_base_*).

$$\mathrm{GDD}=\frac{\sum_{t=0}^{23}T_{t}}{24}-T_{\text{base}}$$ (1)

where *T_t_* is hourly temperature of the day. *T_base_* was set to 10 according to the previous study [32]. Since DeepCrop was not extremely fine-tuned, other types of GDD were not considered. Vapor-pressure deficit (VPD) was calculated from saturation vapor pressure (SVP) using temperature and relative humidity (Eqs. 2 and 3).

$$\begin{array}{c} \mathrm{SVP}=0.6113\times \exp\left(5,423\left(\frac{1}{273.15}-\frac{1}{273.15+T}\right)\right) \end{array}$$ (2)

$$\mathrm{VPD}=\mathrm{SVP}\left(1-\frac{\mathrm{RH}}{100}\right)$$ (3)

where *T* is greenhouse temperature (°C) and RH is relative humidity (%).

### Experimental and technical design

As mentioned in the Introduction section, DeepCrop was developed to become substitutional for the existing crop models. To satisfy this requirement, the attention mechanism was used for the core algorithm of DeepCrop [24]. Attention mechanisms are a full-neural-network algorithm that has shown high performance in interpreting the sequence data, and it can also be applied to various data types, such as text and image [33,34]. Therefore, the mechanism could be suitable for application to crop growth that is highly affected by time.

**Fig. 1.**
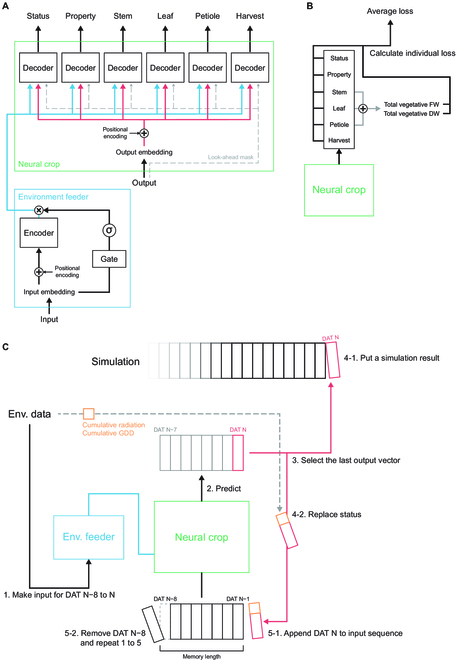
Modeling and simulation workflows. (A) Model structure. In this study, multitask decoders and an input gate were added to the original encoder and decoder of the original transformer algorithm. Refer to the Supplementary Materials for the detailed structure of the embedding layer. (B) Target loss objective of the model training. In this study, the loss function was the mean squared error. (C) Simulation procedure of the trained DeepCrop. The numbers represent the operation order.

However, the original attention mechanism is specialized to the natural language processing, so some modifications were essential (Fig. 1A). The algorithm uses a Word2Vec embedding layer, but the word space must be trained with the enormous sentences first [35]. In this study, the embedding layers were substituted by the convolutional layers. In addition, the attention algorithm supposes input sentences with an arbitrary length, so the model requires relevant structures such as padding masks and end tokens. DeepCrop was constructed to be fed input with a fixed length; thus, these structures were eliminated. Lastly, DeepCrop had multiple types of the output; hence, it had multiple decoders to interpret each organ. DeepCrop also had modifications in the model loss function (Fig. 1B). The output from each decoder was separately averaged, and the losses were calculated for a stable simulation. Additionally, some obvious facts could improve convergence stability. That is, the weight summation of the total vegetative organs should be equal; thus, the losses of the total vegetative DW and FW were also considered. Therefore, DeepCrop minimized the average value of the 6 calculated losses from the decoders and 2 additional losses. Crop environment and growth data were fed to the model for training, and the trained DeepCrop obtained daily environment and initial growth factors for a simulation (Fig. 1C).

### DeepCrop structure

**Fig. 2.**
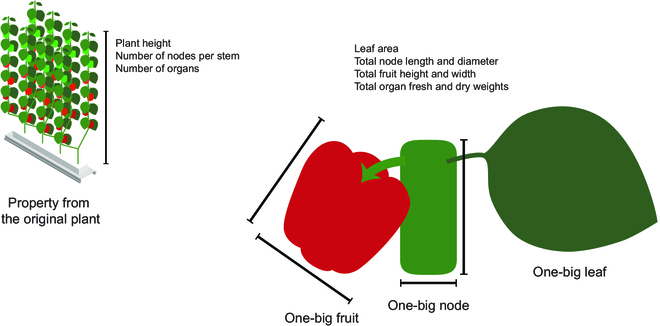
Modeling concept. Target crop growth and morphology were abstracted as one-big organs. Averages can be calculated with total values and the number of organs.

The model was designed to predict the growth and harvest per plant from the crop environment (Table 2). For the simulation, target crop growth and morphology were abstracted as one-big organs. Since sweet peppers have several organs for a long time, average growth factors could be diluted with the fully grown organs; therefore, the simulation was based on the total values, not on the average ones. The average growth factors can be calculated with total values and the number of organs. Some growth factors, such as plant height, that cannot be inferred from the total values were from the original plant (Fig. 2).

**Table 2.** Input and output of DeepCrop. Organ represents leaf, stem, petiole, and harvested fruits. In the same day, daily values of the input were the same for 24-h values.

Group	Factor
Input	Internal temperature (°C)
	Internal relative humidity (%)
	Radiation (W m^−2^)
	Daily difference between the day and night temperature (°C)
	Daily cumulative radiation (kJ m^−2^ day^−1^)
	Cumulative growing degree days
	Calculated daily vapor-pressure deficit (kPa)
Status output	Total cumulative radiation (MJ m^−2^)
	Cumulative growing degree days
Property output	Plant height (m)
	Maximum number of nodes per stem
Organ output	Leaf area (m^2^)
	Total summation of node lengths (cm)
	Total summation of node diameters (mm)
	Total summation of harvested fruit heights (cm)
	Total summation of harvested fruit widths (cm)
	Number of organs
	Organ fresh weight (g)
	Organ dry weight (g)

The attention mechanism can interpret complicated sequence data with faster computation [33]. DeepCrop was mostly based on Transformer, and the core algorithms, such as positional encoding, look-ahead mask, and multihead attention, were the same [24]; however, since the main task for Transformer was classification, some structures had to be modified (Figs. S1 and S2). The modified structures were inspired by the difference that the object is not linguistic sentences but sets of concrete numerical values. The data in this study can be calculated with each other if necessary; therefore, embedding is designed not to reduce the dimension of the input, but to expand it to contain diverse features. In this regard, input and output were embedded by concatenating convolutional layers with different receptive fields to mimic the window in Word2Vec [36]. The embedding layers were required to match the dimension of internal data processing. Therefore, input and output embedding layers had the same number of nodes. A gate for residual calculation was added because raw input before the encoding is also important for the target output.

Multitask decoders were added to predict each target group. The decoders had the same structure for applicability. Transformer can deal with the sequences regardless of the input and output length; however, the number of cultivations was limited; thus, the model should partially receive and predict the environment and growth. Therefore, the input and output length were fixed with a parameter named memory length. The output dimensions of the multitask decoders were set to be same with the memory length. The unit of the memory length is supposed to be days; hence, the input length was determined as 24 times of the memory length. The dimension of the datasets was matched to be the same based on the batch size and the memory length. Therefore, DeepCrop always processed the same dimension of data; thus, the padding mask of the attention mechanism was not used in this study. Meanwhile, the positional encoding was required to mark the position of each input and output vector, although the data dimension was fixed.

The reasoning of DeepCrop was tested after the model training using 2-dimensional t-distributed stochastic neighbor embedding (t-SNE) and attention weights. Both methods are generally used to explore the black-box condition of the deep learning models [37,38]. Some physiological tendencies, such as developmental stages, were verified.

The loss function was set to the mean squared error (MSE). The MSE of each decoder was independently calculated. To maintain the relationships among the decoders, the total FW and total DW of vegetative organs were also compared. All MSEs were averaged and traced in the model training session.

### Data preprocessing for the model training

**Fig. 3.**
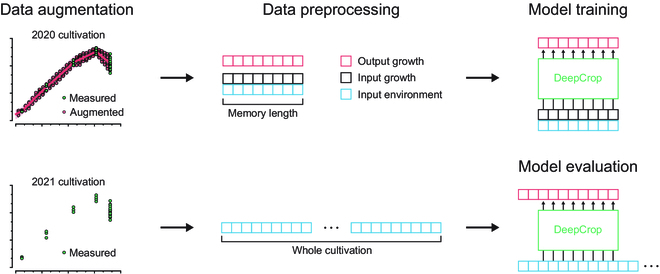
Data processing sequence. Refer to Fig. 1 for the detailed DeepCrop structure.

Since the training process of DeepCrop was based on supervised learning, daily growth data in the cultivation periods had to be secured as labels; therefore, scarce growth data should be augmented. Using the output of regression or an existing formula is a way to augment the data, but it could affect the model results and lower the model accessibility. In this study, the scarce growth data and their standard deviations were interpolated linearly, and the daily output was randomly augmented (Fig. S3). The start date of the cultivation was set to 50% of the first growth data. The model was trained and evaluated with a somewhat complicated process for the general robustness with the limited number of datasets (Fig. 3).

### Training, validation, and evaluation process

The core algorithm of DeepCrop was the attention mechanism; thus, the model training and evaluation process differed. At the training session, DeepCrop was fed sets of environment data, previous growth factors, and the target output. Since the previous growth factors do not simultaneously exist in a practical simulation, the DeepCrop output recursively replaced previous growth factors. The last output vector of the output sequence was selected as the daily predicted output. The data from 2020 were used for the training and validation datasets, and those from 2021 were used for the test datasets. Cumulative temperature and radiation can be calculated with the environment data; therefore, the cumulative input factors were replaced by the measured data in practice to guide the trained DeepCrop; however, the values were not replaced to prevent training failure in the model training. The number of the data in the training, validation, and test datasets were 18,900, 8,100, and 254, respectively.

### Existing crop models and deep learning models for comparison

Some accessible crop models were compared as an existing method: a simple generic crop model (SIMPLE) [39], a sweet pepper growth model for a decision support system (SW-DSS) [40], food and feed crop models from World Food Studies (WOFOST) [9], and a sweet pepper model of Decision Support Systems for Agrotechnology Transfer (DSSAT) [41]. Some models were modified for comparison. The SIMPLE model yields total dry mass, including reproductive organs; in this study, the value was changed to vegetative dry mass with a harvest index of >1. SW-DSS had an ambiguous LAI equation; hence, the equation changed to Eq. 4 based on the definition of the Gompertz growth function [42].

$$\begin{array}{c} {\mathrm{LAI}}_{t}={\mathrm{LAI}}_{max}\exp\left(\log\left(\frac{{\mathrm{LAI}}_{\text{init}}}{{\mathrm{LAI}}_{\max}}\right)\exp\left(\;-C_{\text{pLAI}}\times \mathrm{TS}\right)\right) \end{array}$$ (4)

where *LAI_max_* is the maximum LAI, *LAI_init_* is the initial LAI, *C_pLAI_* is a tuning parameter of the Gompertz function, and *TS* is the thermal time of the plant (°C per day). SW-DSS originally had a temperature-dependent gamma star for the photosynthesis calculation, but it was also calibrated as a coefficient in this study.

Modeling efficiency (EF) and normalized root mean squared error (NRMSE) were selected to compare DeepCrop and the other crop models [7,43] (Eqs. 5 and 6).

$$\mathrm{EF}=1-\frac{\sum_{i=1}^{n}{\left(y_{\text{label},i}-y_{\text{pred},i}\right)}^{2}}{\sum_{i=1}^{n}{\left(y_{\text{label},i}-y_{\text{label},\text{mean}}\right)}^{2}}\;$$ (5)

$$\text{NRMSE}=\frac{\text{RMSE}}{y_{\text{label},\max}-y_{\text{label},\min}}$$ (6)

where *y_label_* and *y_pred_* represent measured and predicted target growth factors, respectively. EF = 0 represents that the evaluated model yields the same performance as simply averaging the observed data would; EF = 1 represents a perfect match of observed and simulated values. Since LAI and fruit yield, the target growth factors, could be similar in all greenhouses, EF can emphasize differences in calibrated models.

Since no model could cover every output of DeepCrop, the performance was calculated with the partial output, and the units of model output were converted as per plant, similar to DeepCrop. Petiole FW and DW were aggregated to those of stems when the crop models could not calculate petioles independently. The leaf area index was converted to the leaf area using planting density for the models that calculated only the leaf area index. Planting density was adjusted for the crop models that were completely biased because of the conversion (Table 3). All models assumed sufficient irrigation without water stress.

**Table 3.** Adjusted planting density of 2020-2 for the compared crop models.

Crop model	Value
Original	5.95
DSSAT	2.00
WOFOST	5.95
Sánchez-Molina et al. (2015)	2.56
SIMPLE	3.70

The crop models were calibrated using the data from 2020 that were the same as the training and validation data of DeepCrop (Tables S1 to S4). The sweet pepper model of DSSAT was calibrated using Generalized Likelihood Uncertainty Estimation, a built-in coefficient calibrator [44], and a recently calibrated model was also compared [45]; the other models were calibrated using a hyperparameter optimizer algorithm, HyperOpt [46].

As a shallow deep learning model, a feedforward neural network, long short-term memory (LSTM), and convolutional neural network were compared. The models are representative structures of deep learning algorithms. Since the shallow models cannot efficiently interpret the same DeepCrop input and output, the optimal data structure for each model was applied (Table S5). The deep learning models were set as a predictor, not a process-based model; therefore, the growth factors were not recursively reprocessed by the models. Hyperparameters for the model construction and training were empirically selected (Table S6). The average of the growth factors in 2020 was used as a baseline.

### Computations

AdamOptimizer was used for the training of deep learning models [47]. Batch and layer normalizations were used for regularization [48,49]. A deep learning library in Python, TensorFlow (v. 2.6.0), was used to build the model [50]. All deep learning computations were conducted using a Linux server with one central processing unit (CPU) (ThreadRipper 2990WX, AMD, Santa Clara, CA) and one graphics processing unit having 35.58 TFlops (RTX 3090, NVIDIA, Santa Clara, CA). Non-deep-learning crop models were simulated only with CPUs.

## Results

### Simulated crop growth from DeepCrop

**Fig. 4.**
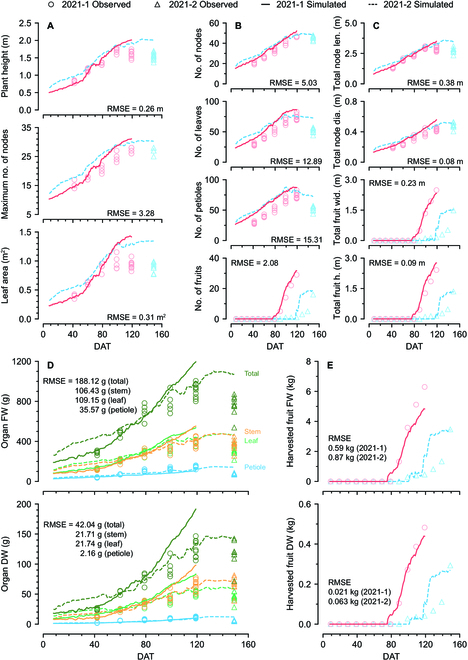
Simulation results from DeepCrop. (A) Property output and leaf area. Leaf area was predicted by a leaf decoder. (B) Simulated number of organs. (C) Total summation of node lengths, node diameters, harvested fruit widths, and harvested fruit heights. (D) FW and DW of the vegetative organs. (E) Cumulative FW and DW of the harvested fruits. All data represent the value per plant.

Trained DeepCrop was evaluated with the data from the first and the second halves of 2021, represented as 2021-1 and 2021-2, and it showed reasonable simulations for the test data (Fig. 4). The output tendencies varied, although the model shared the same encoder. Some simulation results showed a declining section in the middle of the cultivation. DeepCrop somewhat overestimated the vegetative growth factors. The tendency was obvious for the FWs of 2021-2. For the harvested fruits, the simulated factors of 2021-1 were similar to the observed values, and those of 2021-2 were not; however, the final FW and DW of harvested fruits could be accurately predicted. Overall, the simulation reasonably followed the tendency of the observed growth for the diverse target factors without considering the interactions of each factor and relevant formula.

### Model evaluation and comparison

**Fig. 5.**
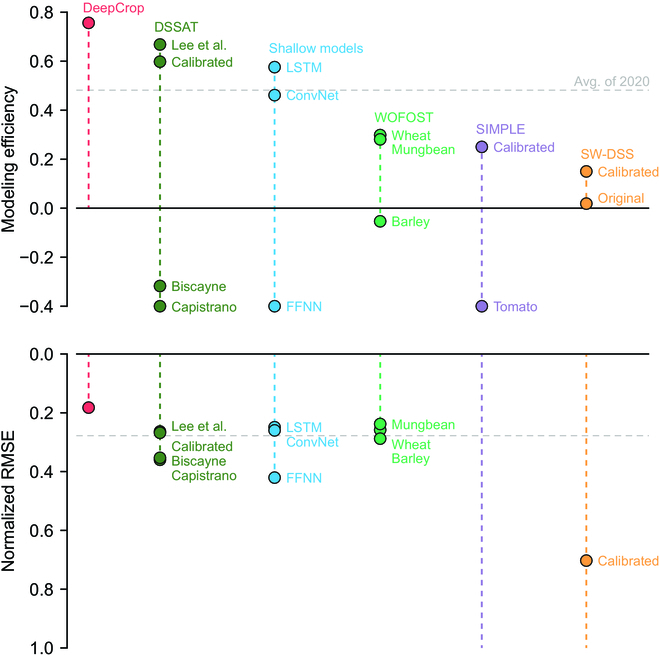
Model performance of existing models. The modeling efficiency and normalized root mean squared error (normalized RMSE) were calculated with the data from 2021-2. FFNN, LSTM, and 1D ConvNet represent feedforward neural network, long short-term memory, and one-dimensional convolutional neural network, respectively; WOFOST, DSSAT, SIMPLE, and SW-DSS represent World Food Studies [9], Decision Support System for Agrotechnology Transfer [41], a simple crop model [39], and a sweet pepper model for a decision support system [40], respectively. For WOFOST, the top 3 calibrated models were depicted. The normalized RMSEs out of the axis boundary were omitted.

DeepCrop was compared with existing accessible process-based crop models and simple predictors based on deep learning algorithms. Modeling efficiency (EF) and normalized mean squared error (NMSE) showed different tendencies; however, DeepCrop recorded the highest EF and the lowest NMSE compared to the other accessible models, although advanced horticultural models such as HORTISIM could not be listed (Fig. 5). According to EFs, food and feed crop models were not effectively calibrated for sweet pepper, although the targets were limited to organ DWs; however, 2 calibrated models recorded the lowest NMSE among the compared models. The crop models that initially targeted sweet pepper were also unable to simulate the given data except the model in DSSAT. The calibrated DSSAT models showed competitive performance; however, all existing crop models needed adjusted plant density for the unit conversion from the original to value per plant.

For the deep learning models, LSTM showed the highest performance, but it was not simply comparable because the deep learning models were not process-based. Specific results of the compared models are given in Figs. S4 to S6.

**Fig. 6.**
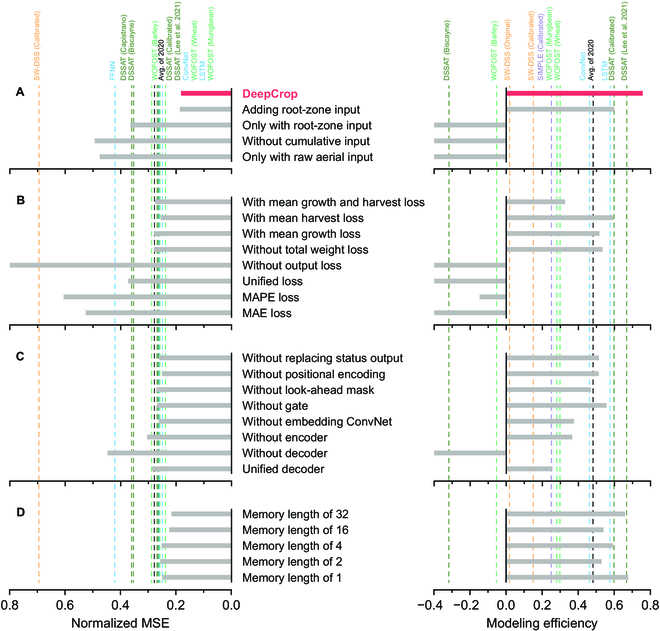
DeepCrop with ablation. The dashed lines are the accuracy of the corresponding models. The ablation test was conducted for variations in (A) input, (B) training procedure, (C) model structure, and (D) memory length. The results outlying the depicted axis are not shown.

According to the ablation test, adequate selection of the input features was the most deterministic factor of DeepCrop performance (Fig. 6A). All input variations were not adequate for the simulation; in particular, adding more information did not ensure higher performance. Providing cumulative input and manipulated input were effective for the model performance. For the loss objectives, dividing the loss for each decoder made the model converge well (Fig. 6B). Domain knowledge, such as the loss of total FW and DW, was also effective for model robustness. Similar to the input variation, more loss objectives based on trivial plant physiology did not ensure model convergence. All structural ablations decreased the model performance, and the existence of the decoders was the decision factor (Fig. 6C). Excluding the original attention mechanism and modified algorithms also decreased model stability. Increasing the memory length that determined the output length for the decoders did not guarantee an improvement in model performance (Fig. 6D).

**Fig. 7.**
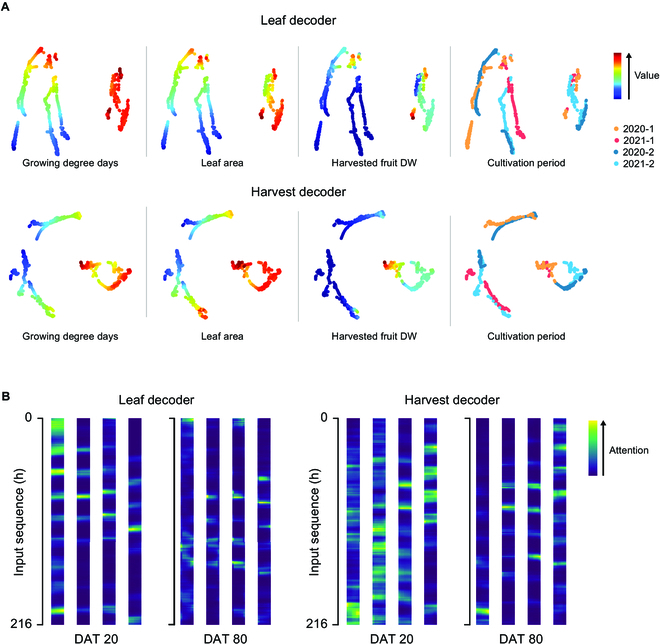
(A) Two-dimensional t-distributed stochastic neighbor embedding (t-SNE) for the output of the last hidden layer and (B) attention weights yielded by the Leaf and Harvest decoders. Fully-trained DeepCrop was used to yield the output. Two decoders were selected as representatives of the vegetative and reproductive representations. Attention weights were extracted from the DAT 20 and 80 to represent the early and later part of the cultivation, respectively. The columns of the attention weight yielded from each head in the attention mechanism. The number of the columns represents the number of the attention heads. Only 2021-1 data were used for the attention weights.

Two-dimensional t-SNE showed 2 distinctive clusters (Fig. 7A). Environment and growth factors did not perfectly reflect the division; however, the factors were largely divided into high and low values. Four cultivations did not have completely different distribution, but the harvest decoder showed distinct distributions of harvested fruit DW for the first and second half of the year. The input sequences were utilized in balance according to the attention weights (Fig. 7B). The attention tendency of Leaf and Harvest decoders were different. In the latter part of the cultivation, both attentions were relatively narrowed.

## Discussion

The attention mechanism with multitask decoders was able to interpret the interactions of crop and environment with a high performance. It has been reported that sharing root layers and dividing the tasks that have similarities but differ in the final output can improve performance [51,52]. Since all the target output were trained well, the normalization strategies such as Batch and Layer Normalization in DeepCrop seemed to be adequately worked. Therefore, multitask decoders could be the core structure. In this study, the neural network models with shallow structures also showed acceptable scores. Theoretically, it has been verified that one layer of neural networks can interpret all forms of functions [53]. Finding a superficial relationship between the environment data and the crop growth using regression methodology is a relatively simple target for deep learning algorithms; thus, shallow models can achieve high performance if the models are technically well fitted. However, the compared deep learning models did not receive crop growth as input; that is, the models were a predictor, not a process-based crop model. Therefore, shallow models cannot interpret large datasets with several differences in crops, scales, and so on. In addition, Transformer is faster in calculation and can have a deeper structure [24], and the performance gap between LSTM and DeepCrop could increase with the amount of data.

Understandably, sweet peppers in greenhouses could have a large disjunction with food and feed crop models for open fields. Crop models in WOFOST share the same physiology module [9,54], but it seems that the module has a substantial difference from sweet peppers. However, the original sweet pepper models also failed to simulate the test data. It was impossible to compare the existing models without adjusted plant density because of the low accuracy even after the calibration. Therefore, it can be said that the functions in crop models are overfitted to their dataset and research scales. Crop model ensembles or simplified estimation factors could improve simulation performance [7,17,55]; however, the methodologies cannot be a fundamental solution for overfitting, that is, fragmentation of the crop models.

For DeepCrop, the model selection and training did not require meticulous understanding of the plant physiology and crop modeling; that is, consideration of suitable functions and coefficients were not essential. Existing crop models must modify the innate functions if the model robustness cannot be secured with calibration for new data [32,55,56]. On the other hand, the same deep learning models can be retrained for the new task in any case [57,58]; even if a model with better performance appears, that model also has the same properties under deep learning. Therefore, DeepCrop can be retrained using the same methodology, including a new dataset, and it can improve the accessibility to crop models. In the same context, DeepCrop can also easily be tuned to interpret other new technologies such as hyperspectral imaging and computer vision, although the model was trained only with the aerial environment for the comparison.

DeepCrop simulation adequately followed the growing tendency from scratch according to the scores, but the model should be inspected because it has potential to be improved. Specifically, DeepCrop overestimated the test data despite the high score. This seems to be due to the difference in management. Crop growth was somewhat insufficient because of some inhibitory factors, such as high planting density and unfavorable weather; therefore, fruiting was deliberately delayed, which resulted in an inverse tendency of cumulative harvested fruits. That is, the data were not sufficient to perfectly interpret hidden patterns in crop growth; hence, DeepCrop depended on the last cultivation too much.

However, the model was adequately trained according to the simulation result. This yielded different results for 2021-1 and 2021-2. The model recognized seasonal differences that were difficult to grasp with only a given input. Similar to the other models, it was impossible to fully understand the phenology of sweet pepper with 4 cultivation datasets. Nevertheless, the model was adequately trained with limited conditions; therefore, the simulation result concretely showed the potential of DeepCrop for larger datasets. More types of data will enable the identification of clear growth patterns and an understanding of crops without human knowledge [27]. DeepCrop is still a black box model, but the studies to reveal the reasoning process are ongoing [59,60]; DeepCrop can learn the intermediate output such as assimilation from a sufficient number of data and features, and it can also make the model explainable [61].

The t-SNE distribution and the attention weights also support that the recognition of the growth pattern of DeepCrop can be analyzed out of the black box. DeepCrop could interpret the relationship between the environment and growth without being biased to a certain factor. Four cultivations did not show completely different distributions; the developed model was not biased to a certain cultivation period. In particular, 2 clusters can be regarded as the division of the vegetative and reproductive stage. The attention weights showed different tendencies by the crop development stage and the output decoder. Differences by input factors were also shown, although they were relatively weak. The weights could include the detailed information about the environmental influences on crops. Therefore, more datasets would enable the analysis of DeepCrop reasoning.

Currently, since the data are scarce because of the characteristics of crop production, all processes, including input feature selection, model construction, model training, best model selection, and model testing, must be carefully conducted. For the input used in this study, growing degree days, vapor-pressure deficit, and difference between the day and night temperature are often used for crop modeling [9,15,32]. Therefore, utilizing domain knowledge with insufficient data could improve model convergence. However, careless repetition of redundant information can cause overfitting. According to the ablation test, excessive input features in the small dataset caused overfitting, although deep learning can autonomously extract features from raw data. Applying an end-to-end deep learning framework seems to be premature [62]; thus, the balance should be kept in input feature selection.

The training process was also able to influence the robustness in crop modeling. Dividing the loss function by multitask learning was effective for the model performance, but too many loss objectives resulted in counter effects. Gradient descent is linear-algebraically sophisticated [63,64]. Better loss objectives should be found for obvious objectives, such as the number of leaves equal to the number of petioles. Specialization of the tasks and trainable losses with more features can yield a stable and accurate model. For the output setting in the training process, guiding the output with known values also slightly improved the model performance. However, guiding only with Status output was not that effective; hence, more variations of guiding output should be identified.

The discordance in metrics showed the necessity of diversified evaluation for deep learning models that experience difficulty in analyzing the reasoning process. The main problem could be a scarcity of the labels. Finding adequate metrics for scarce data or continuous measuring devices to increase the number of labels should be introduced.

## Conclusion

In this study, a process-based crop model that was fully constructed with deep learning algorithms was developed and evaluated. DeepCrop consists of an attention mechanism and multitask decoders. Trained DeepCrop showed the highest accuracy in selected metrics, modeling efficiency and NRMSE. With the precedents in the other research fields, the model can be trained only with raw data without domain knowledge. DeepCrop does not require consideration of the internal formula corresponding to the input variation; thus, the same structure can be applied to diverse studies unless the target task is identical. In this study, advanced horticultural models were not compared; since the model structure and the relevant workflow are explained, further studies that compare DeepCrop to those models can be conducted. Therefore, we expect that the developed DeepCrop can improve the accessibility of crop models and mitigate fragmentation problems in crop model studies.

## Data Availability

All data and codes are available upon reasonable request.
