# Supplementary material for: Process-Based Crop Modeling for High Applicability with Attention Mechanism and Multitask Decoders
Source: Plant Phenomics. 2023 Apr 12;5:0035. doi: 10.34133/plantphenomics.0035 (PMC10202189; doi:10.34133/plantphenomics.0035)
Supplement: Supplementary 1 — Tables S1-S6 Figs. S1-S6 [file plantphenomics.0035.f1.docx]

**Process-based crop modeling for high applicability with attention mechanism and multitask decoders**

Taewon Moon^1,3^, Dongpil Kim^2^, Sungmin Kwon^1^, Jung Eek Son^1,3,*^

^1^Department of Agriculture, Forestry and Bioresources, Seoul National University, Seoul 08826, Republic of Korea

^2^Protected Horticulture Research Institute, National Institute of Horticultural & Herbal Science, Rural Development Administration, Haman 52054, Korea

^3^Research Institute of Agriculture and Life Sciences, Seoul National University, Seoul 08826, Republic of Korea

*Correspondence author. Email: sjeenv@snu.ac.kr

Supplementary Table 1. Calibrated coefficients of WOFOST. The coefficient values with colons represent the values for the corresponding variables, such as development stage or temperature.

| Item | Description | Value | | |
| --- | --- | --- | --- | --- |
|  |  | Wheat | Mung bean | Barley |
| TEFFMX | Maximum effective temperature for emergence | 100 | 100 | 100 |
| TBASEM | Base temperature for emergence | 0 | 0 | 0 |
| TSUMEM | Temperature sum from sowing to emergence | 0 | 0 | 0 |
| SPAN | Life span of leaves growing at 35 Celsius | 400 | 400 | 400 |
| DVSI | Initial development stage at the start of simulation | 0.48 | 0.63 | 0.89 |
| TBASE | Lower threshold temperature for ageing of leaves | 21.37 | 11.39 | 14.54 |
| RGRLAI | Maximum relative increase in LAI | 1.61 | 3.59 | 3.35 |
| TSUM1 | Temperature sum from emergence to anthesis | 676 | 733 | 713 |
| TSUM2 | Temperature sum from anthesis to maturity | 1402 | 1200 | 1274 |
| CVO | Conversion efficiency of assimilates into storage organ | 0.45 | 0.91 | 0.94 |
| CVL | Conversion efficiency of assimilates into leaf | 0.85 | 0.51 | 0.36 |
| SLATB | Specific leaf area as a function of development stage | 0: 0.026  1: 0.014  2: 0.019 | 0: 0.030  1: 0.043  2: 0.014 | 0: 0.035  1: 0.021  2: 0.023 |
| SSATB | Specific stem area as a function of development stage | 0: 0.054  1: 0.051  2: 0.011 | 0: 0.012  1: 0.036  2: 0.001 | 0: 0.042  1: 0.031  2: 0.001 |
| KDIFTB | Extinction coefficient for diffuse visible light as function of development stage | 0: 0.813  1: 0.129  2: 0.817 | 0: 0.237  1: 0.384  2: 0.636 | 0: 0.179  1: 0.651  2: 0.756 |
| AMAXTB | Maximum leaf CO_2_ assimilation rate as a function of development stage | 0: 35.66  1: 19.10  2: 34.54 | 0: 29.55  1: 33.08  2: 30.26 | 0: 39.95  1: 22.18  2: 24.45 |
| RFSETB | Reduction factor for senescence as function of development stage | 0: 0.085  1: 0.903  2: 0.044 | 0: 0.532  1: 1.000  2: 0.840 | 0: 0.448  1: 0.713  2: 0.906 |
| FRTB | Fraction of total dry matter increase partitioned to roots as a function of development stage | 0: 0.28  1: 0.40  2: 0.07 | 0: 0.20  1: 0.18  2: 0.43 | 0: 0.39  1: 0.03  2: 0.01 |
| FLTB | Fraction of above ground dry matter increase partitioned to leaves as a function of development stage | 0.0: 0.90  0.5: 0.40  1.0: 0.93  1.5: 0.70  2.0: 0.16 | 0.0: 0.57  0.5: 1.00  1.0: 0.29  1.5: 0.05  2.0: 0.29 | 0.0: 0.99  0.5: 0.63  1.0: 0.83  1.5: 0.84  2.0: 0.26 |
| FSTB | Fraction of above ground dry matter increase partitioned to stems as a function of development stage | 0.0: 0.09  0.5: 0.50  1.0: 0.01  1.5: 0.01  2.0: 0.49 | 0.0: 0.16  0.5: 0.00  1.0: 0.30  1.5: 0.27  2.0: 0.32 | 0.0: 0.01  0.5: 0.27  1.0: 0.06  1.5: 0.14  2.0: 0.41 |
| FOTB | Fraction of above ground dry matter increase partitioned to storage organs as a function of development stage | 0.0: 0.01  0.5: 0.10  1.0: 0.07  1.5: 0.29  2.0: 0.35 | 0.0: 0.27  0.5: 0.00  1.0: 0.41  1.5: 0.68  2.0: 0.39 | 0.0: 0.00  0.5: 0.10  1.0: 0.11  1.5: 0.02  2.0: 0.33 |
| EFFTB | Initial light-use efficiency of CO_2_ assimilation of single leaves as function of mean daily temperature | 0: 0.75  10: 1.57  20: 0.27  30: 1.23  40:1.99 | 0: 0.22  10: 1.32  20: 0.92  30: 1.78  40:1.74 | 0: 1.47  10: 1.06  20: 0.23  30: 1.02  40:0.76 |
| DTSMTB | Daily increase in temperature sum as function of average temperature | 0: 25.9  15: 99.9  30: 71.3  45: 87.1 | 0: 63.2  15: 43.6  30: 99.9  45: 64.0 | 0: 52.9  15: 84.7  30: 52.7  45: 43.4 |

Supplementary Table 2. Original and calibrated coefficients of DSSAT.

| Item | Description | Value | | | |
| --- | --- | --- | --- | --- | --- |
|  |  | Biscayne | Capistrano | Lee et al. (2021) | Calibrated |
| EM-FL | Time between plant emergence and flower appearance | 22.0 | 37.0 | 40.0 | 21.99 |
| FL-SH | Time between first flower and first pod | 11.0 | 10.0 | 10.0 | 10.33 |
| FL-SD | Time between first flower and first seed | 15.0 | 15.0 | 15.0 | 15.64 |
| SD-PM | Time between first seed and physiological maturity | 100.0 | 100.0 | 330.0 | 95.80 |
| LFMAX | Maximum leaf photosynthesis rate at 30°C, 350 vpm CO_2_, and high light | 1.10 | 0.98 | 0.98 | 1.071 |
| SLAVR | Specific leaf area of cultivar under standard growth conditions | 250.0 | 275.0 | 275.0 | 299.3 |
| SIZLF | Maximum size of full leaf | 300.0 | 250.0 | 350.0 | 250.0 |
| XFRT | Maximum fraction of daily growth that is partitioned to seed + shell | 0.75 | 0.85 | 0.60 | 0.627 |
| SFDUR | Seed filling duration for pod cohort at standard growth conditions | 25.0 | 25.0 | 40.0 | 25.0 |
| SDLIP | Fraction oil in seeds | 0.050 | 0.050 | 0.050 | 0.051 |
| PM06 | Proportion of time between first seed and physiological maturity that the last seed can be formed | 0.75 | 0.75 | 0.0 | 0.0 |
| FL-VS | Time from first flower to last leaf on main stem | 53.0 | 53.0 | 330.0 | 330.0 |

Supplementary Table 3. Original and calibrated coefficients of a sweet pepper model from Sánchez-Molina et al. (2015).

| Item | Description | Value | |
| --- | --- | --- | --- |
|  |  | Original | Calibrated |
| *Vc_max_* | Maximum carboxylation velocity | 200 | 81.29 |
| *f_c_* | Assimilation conversion factor | 0.29 | 0.30 |
| Γ* | CO_2_ compensation point | Temp. dependent func. | 36.03 |
| *T_ref_* | Reference temperature | 25.0 | 15.1 |
| *r_l_* | Leaf ratio for maintenance respiration rate | 0.030 | 0.004 |
| *r_s_* | Stem ratio for maintenance respiration rate | 0.015 | 0.004 |
| *r_r_* | Root ratio for maintenance respiration rate | 0.015 | 0.001 |
| *r_f_* | Fruit ratio for maintenance respiration rate | 0.010 | 0.002 |
| *T_base_* | Base temperature for thermal time | 10 | 13.86 |
| *VS* | Days of vegetative stage | 30 | 52 |

Supplementary Table 4. Original and calibrated coefficients of SIMPLE.

| Item | Description | Value | |
| --- | --- | --- | --- |
|  |  | Tomato (SunnySD) | Calibrated |
| T_sum_ | Cumulative temperature requirement from sowing to maturity | 2800 | 2931 |
| HI | Potential harvest index | 0.68 | 0.89 |
| I_50A_ | Cumulative temperature requirement for leaf area development to intercept 50% of radiation | 520 | 712 |
| I_50B_ | Cumulative temperature till maturity to reach 50% radiation interception due to leaf senescence. | 400 | 2717 |
| T_base_ | Base temperature for phenology development and growth | 6 | 17.7 |
| T_opt_ | Optimal temperature for biomass growth | 26 | 29.6 |
| RUE | Radiation use efficiency (above ground only and without respiration) | 1.00 | 0.56 |
| T_heat_ | Threshold temperature to start accelerating senescence from heat stress | 32 | 39.6 |
| T_ext_ | The extreme temperature threshold when RUE becomes 0 due to heat stress | 45 | 41 |
| S_CO2_ | Relative increase in RUE per ppm elevated CO_2_ above 350 ppm | 0.07 | 0.02 |
| fSolar_max_ | The maximum fraction of radiation interception that a crop can reach | 0.95 | 0.67 |

Supplementary Table 5. Structure of FFNN, LSTM, and ConvNet. Dense and Conv are a fully connected layer and a convolution layer, respectively; Maxpool and Flatten represent the maximum pooling and flattening, respectively. Parameters for Conv are denoted as “{type of layer}{kernel size}-{number of filters},” and parameters for the other layers are denoted as “{type of layer}-{number of nodes in the layer}.” ResBlock represents a residual block. Refer to Supplementary Fig. 1. for the detailed structure.

| Model | FFNN | LSTM | 1D ConvNet |
| --- | --- | --- | --- |
| Input size | 216×7 | | |
| Layers | Dense-256 | BiLSTM-256 | Conv7-32 |
|  | BatchNorm | LayerNorm | BatchNorm |
|  | Dense-256 | BiLSTM-256 | MaxPool |
|  | BatchNorm | LayerNorm | ResBlock-16 |
|  | Flatten | Dense-32 | ResBlock-32 |
|  | Dense-19 | Dense-19 | ResBlock-32 |
|  |  |  | ResBlock-64 |
|  |  |  | ResBlock-128 |
|  |  |  | Flatten |
|  |  |  | Dense-16 |
|  |  |  | BatchNorm |
|  |  |  | Dense-16 |
|  |  |  | BatchNorm |
|  |  |  | Dense-19 |
| Output size | 1×19 | | |

Supplementary Table 6. Parameters used for each model construction and training. Hyphens represent unused values for the corresponding model.

|  | Value | | | |  |
| --- | --- | --- | --- | --- | --- |
| Hyperparameter | FFNN | LSTM | 1D ConvNet | DeepCrop | |
| Nonlinearity function | Tanh; Sigmoid | Tanh; Sigmoid | ReLU | ReLU; Sigmoid | |
| Normalization | Batch | Layer | Batch | Batch; Layer | |
| Batch size | 128 | 128 | 128 | 256 | |
| Kernel initializer | - | - | Glorot normal | - | |
| Padding | - | - | Same | - | |
| Learning rate | 0.001 | 0.002 | 0.0015 | 0.0006 | |
| Epsilon | 1e-08 | 1e-08 | 1e-08 | 1e-06 | |
| β_1_ | 0.9 | 0.9 | 0.9 | 0.9 | |
| β_2_ | 0.999 | 0.999 | 0.999 | 0.999 | |
| Learning rate decay | 0.1 | 0.1 | 0.1 | 0.1 | |
| Attention heads |  |  |  | 8 | |
| Number of layers |  |  |  | 2 | |
| Memory length |  |  |  | 8 | |
| Embedding dimension |  |  |  | 128 | |


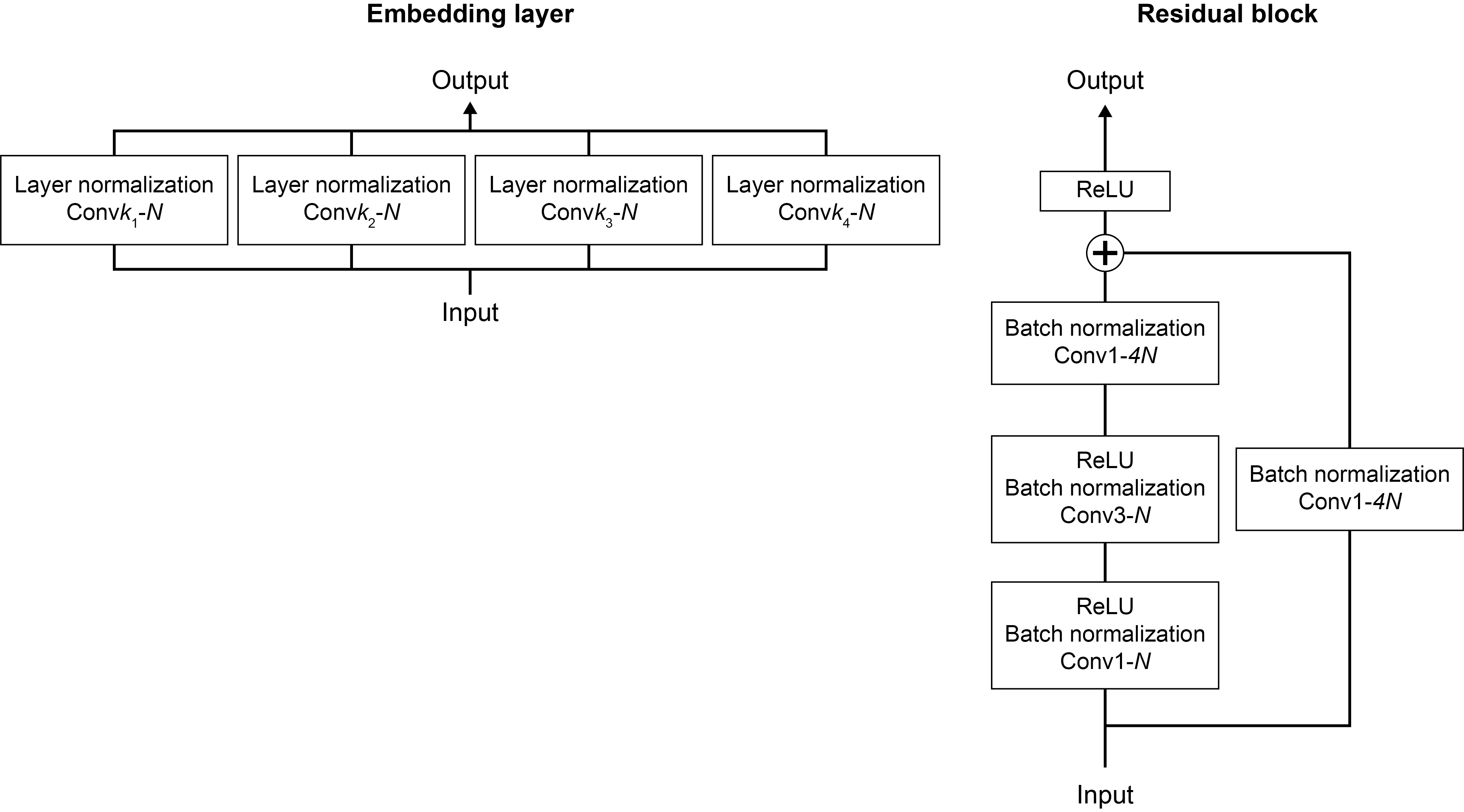


Supplementary Figure 1. Embedding layer for DeepCrop and residual block used for the 1D ConvNet model. Parameters for Conv are denoted as “{type of layer}{kernel size}-{number of filters}.” N was set before the training as a hyperparameter. *k*_1_ to *k*_4_ for input were 1, 12, 24, and 48; those for output were 1, 2, 3, and 4.


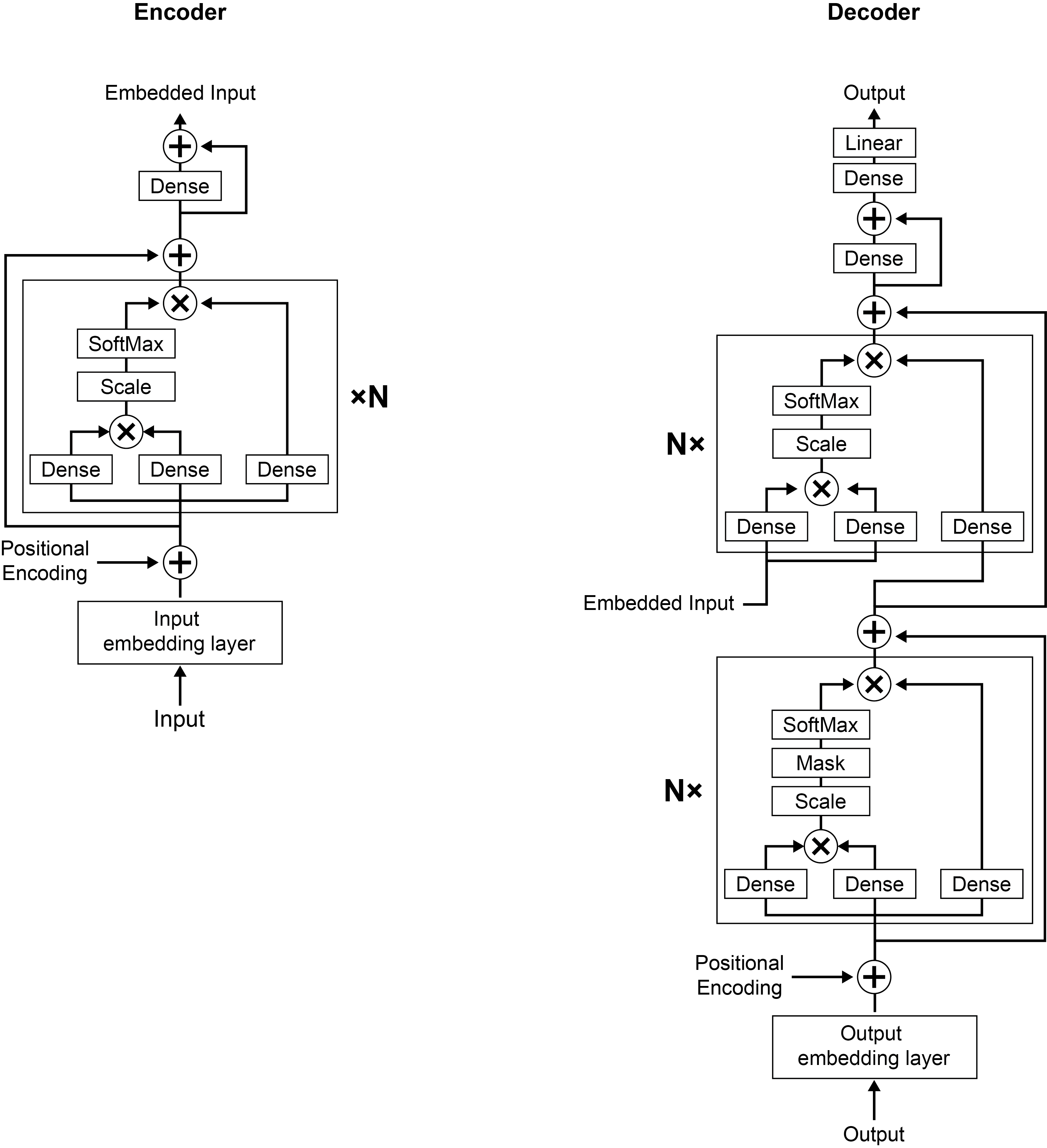


Supplementary Figure 2. Encoder and Decoder for DeepCrop. The symbol N represents the number of head, which was set to four in this study. Dense represents a simple feed-forward neural network without activation functions.

**
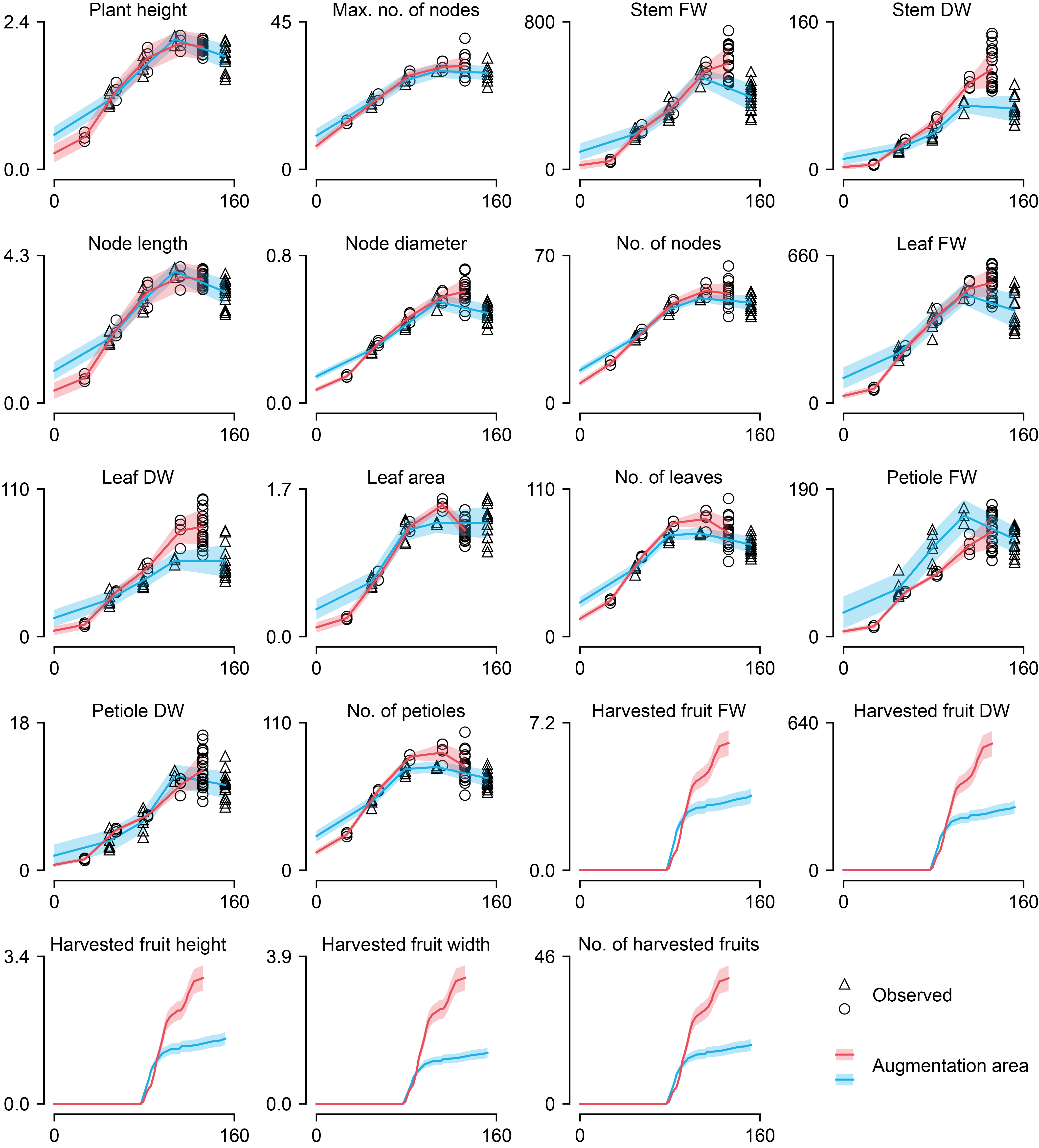
**

Supplementary Figure 3. Distribution of noised growth factors for the model training. Red and blue color represent the first half and the second half of 2020, respectively. FW and DW represent dry and fresh weights, respectively.


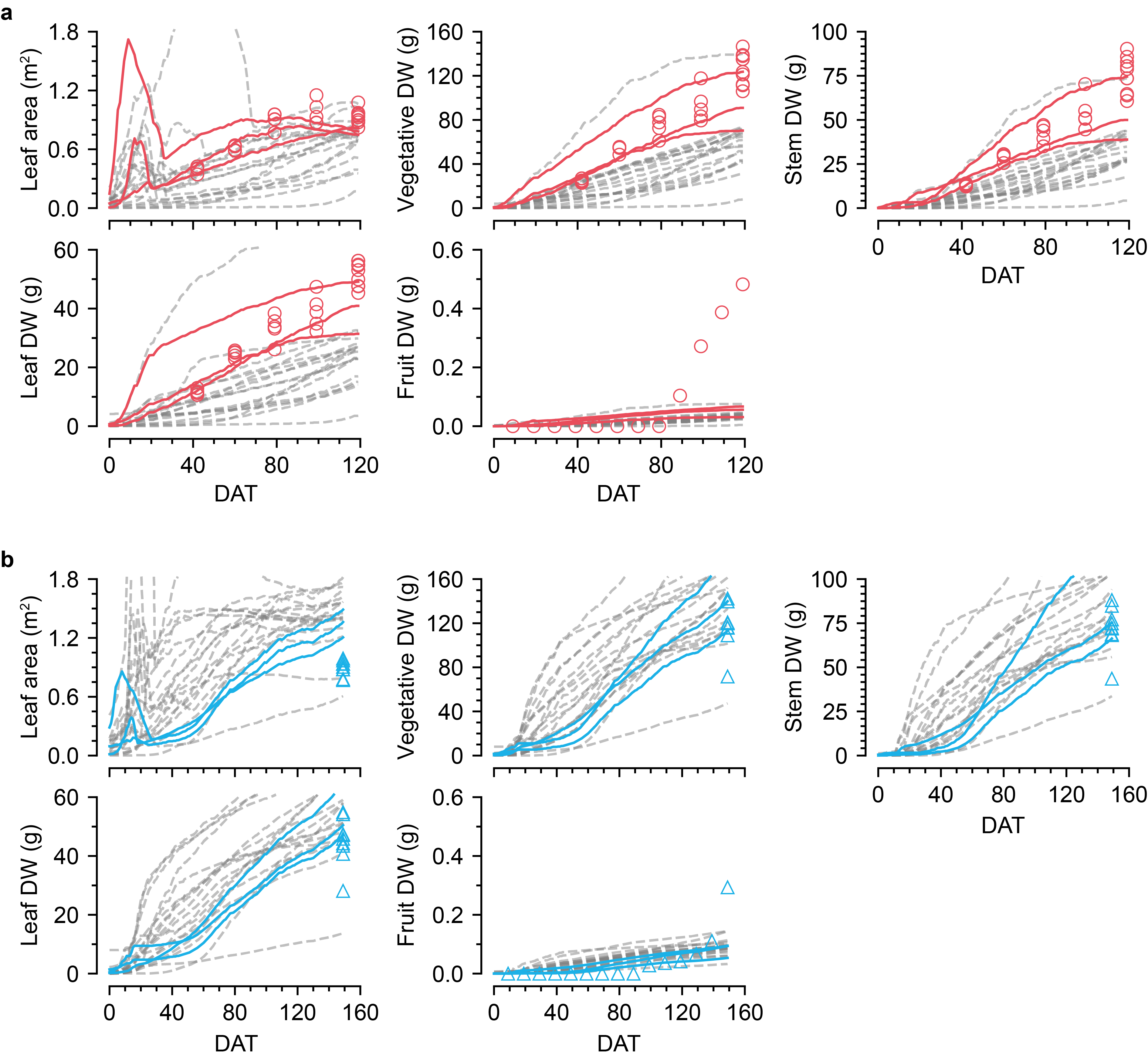


Supplementary Figure 4. Simulated growth factors of calibrated WOFOST crop models for a, 2020-1 and b, 2020-2. Colored solid and gray dashed lines represent the calibrated models that showed the top three performances and the others, respectively. Symbols represent observed growth factors.

**
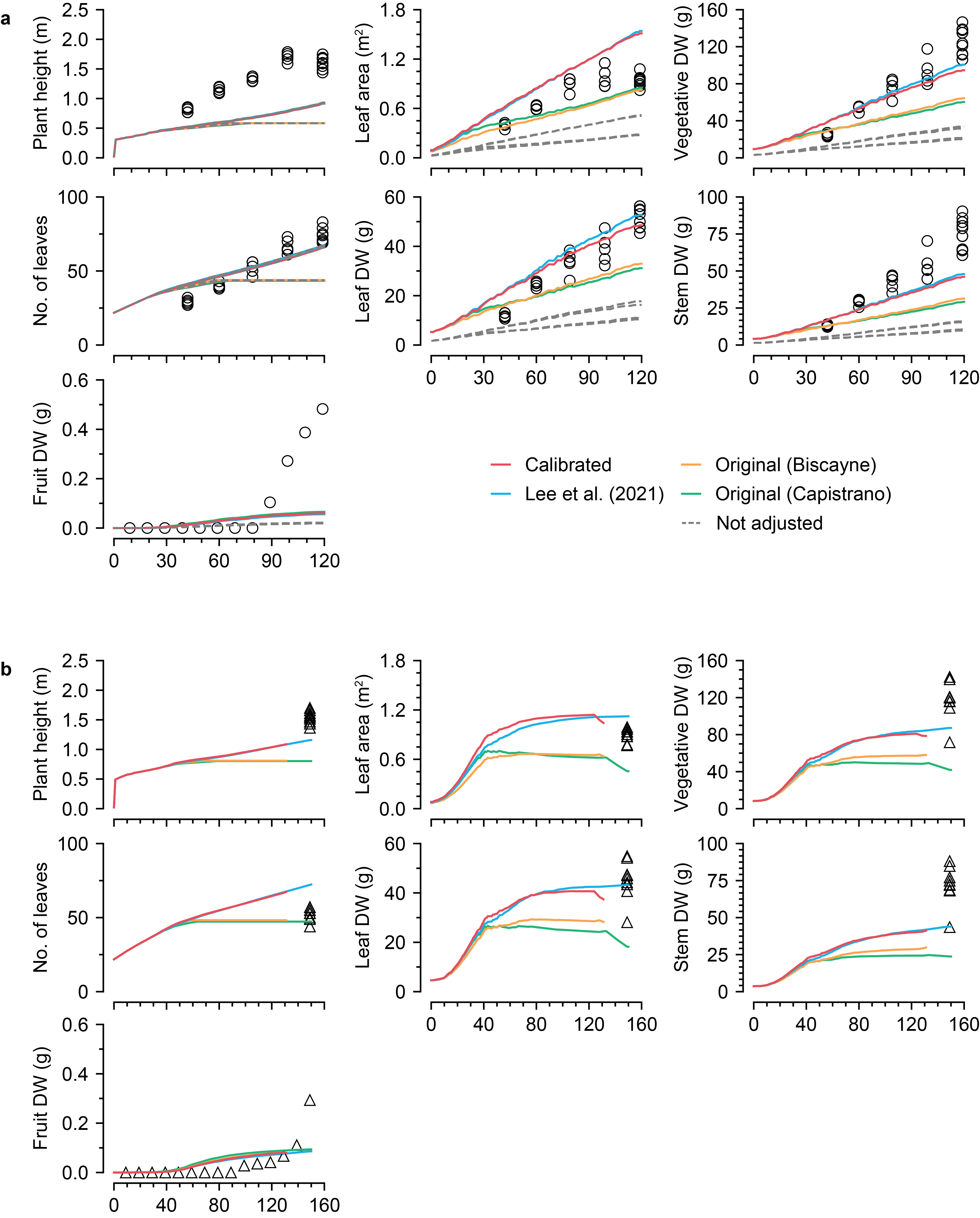
**

Supplementary Figures 5. Simulated growth factors of the sweet pepper model in DSSAT for a, 2021-1 and b, 2021-2. Symbols represent observed growth factors. The gray dashed line represents the simulated 2021-1 without adjusted planting density.


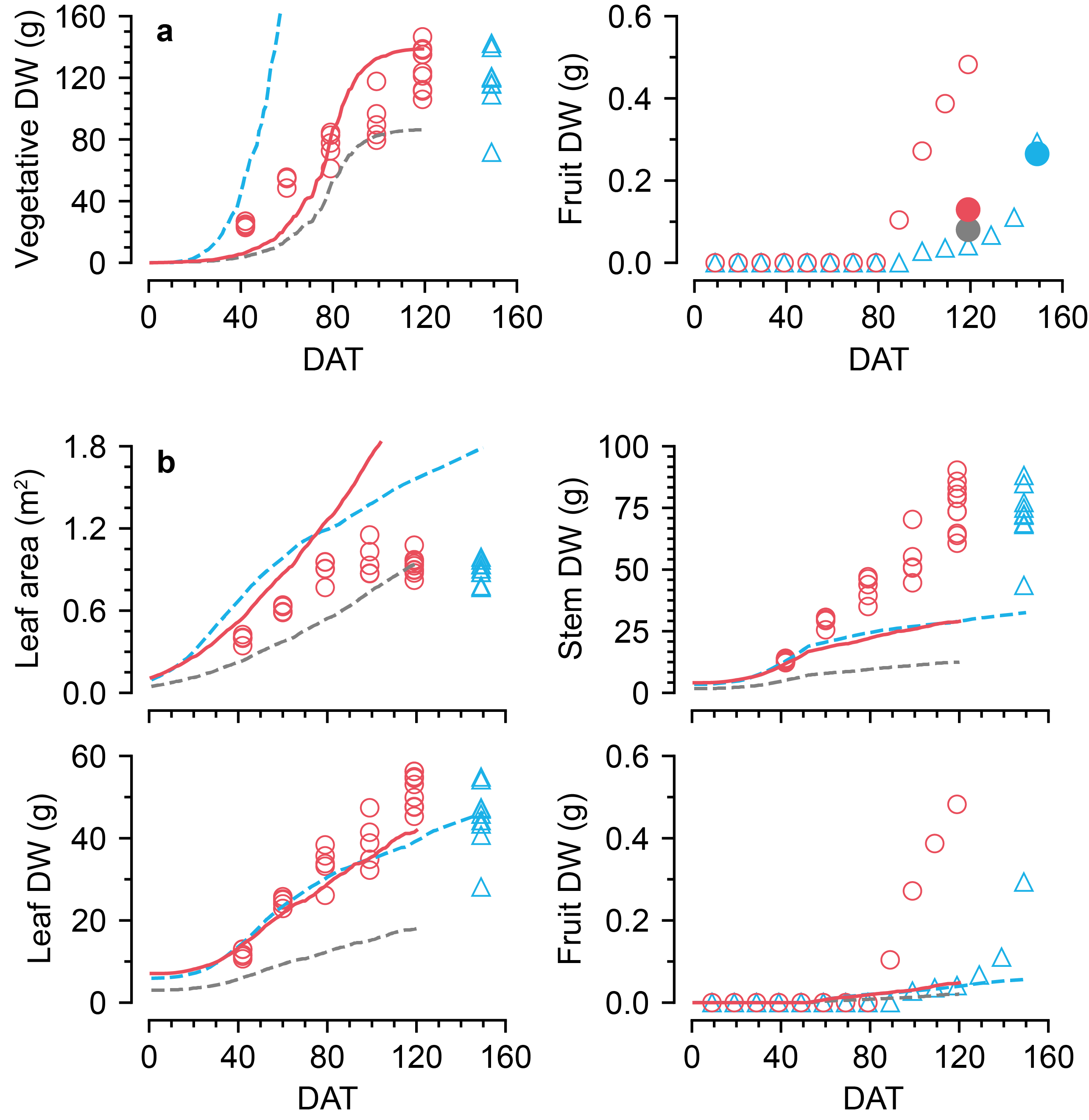


Supplementary Figure 6. Simulated growth factors of a, the SIMPLE crop model and b, the sweet pepper model for decision support Sánchez-Molina et al. (2015).
